# Using Qualitative Behaviour Assessment to Investigate Human-Animal Relationships in Zoo-Housed Giraffes (*Giraffa camelopardalis*)

**DOI:** 10.3390/ani9060381

**Published:** 2019-06-21

**Authors:** Freisha Patel, Françoise Wemelsfelder, Samantha J. Ward

**Affiliations:** 1 School of Animal, Rural and Environmental Sciences, Nottingham Trent University, Southwell NG25 0QF, UK; Freisha.Patel@twycrosszoo.org; 2 Animal and Veterinary Sciences Group, SRUC, Roslin Institute Building, Midlothian EH25 9RG, UK; Francoise.Wemelsfelder@sruc.ac.uk

**Keywords:** animal welfare, human-animal interaction, HAR, keeper-animal interactions, QBA, Free Choice Profiling

## Abstract

**Simple Summary:**

Human-animal relationships can develop from repeated interactions between zoo animals and their keepers. Positive, neutral or negative interactions can result in positive, neutral or negative relationships. Qualitative behavior assessment (QBA) has been utilized as a method to assess emotional expression in animals, but this has yet to be applied to zoo-housed animals. This paper reports a small pilot study aimed at exploring the use of QBA to address human-animal relationships (HAR) in zoos. Three giraffes were video recorded during four different types of keeper-animal interaction producing 38 clips. Using QBA, 18 observers were instructed to describe and score the emotional expressions of the giraffes observed in the clips. In addition, keeper actions during the keeper-animal interactions were assigned positive and negative values by an independent marker, summarized into a final score characterizing their quality. Results indicate that positive keeper actions resulted in calm and confident giraffes with a willingness to interact, whereas negative interactions resulted in more anxious and startled giraffes who were more easily distracted. This suggests that the quality of interaction by keepers influenced the emotional expression of these giraffes, which may affect the giraffe-keeper relationship and have potential welfare implications.

**Abstract:**

Human-Animal Relationships (HAR) in zoos develop from repeated interactions between animals and their caretakers. HAR have been shown to affect health and welfare in farm animals, but limited zoo-based studies exist. This study investigates the association between the qualitative behaviour assessment (QBA) of emotional expression in giraffes and keeper action score in four types of keeper-animal interaction (KAI). Three giraffes generating 38 clips. QBA, using a free-choice profiling methodology, was applied instructing 18 observers to assess giraffe expressions shown in these clips. QBA scores were analysed using Generalized Procrustes Analysis. Keeper actions during each KAI event were rated by an independent marker, resulting in cumulative scores for keeper action quality. The association between QBA and the keeper action was analyzed using Spearman’s rank correlations. Two main QBA dimensions were identified explaining 59% of the variation between clips. There were significant effects of giraffe and KAI type on QBA dimension 2 (inquisitive/impatient—calm/distracted), and significant positive associations between keeper action quality rating and QBA dimensions 1 and 2, indicating that positive keeper actions resulted in calm and confident giraffes with a willingness to interact. This is the first successful application of QBA for empirically addressing HARs in zoos, however given the small sample size of giraffes in this study, it can be regarded as a pilot study only, and further research is needed to validate the use of QBA in this context.

## 1. Introduction

Human-animal relationships (HAR) develop from repeated human-animal interactions (HAI) in agricultural, companion, laboratory and zoo environments [1,2]. Hinde [3] defines an interaction between two individuals as “a sequence in which individual A shows behaviour X to individual B, or A shows X to B and B responds with Y”. Previous studies suggest that repeated interactions between humans and animals in different contexts can lead to the development of a HAR between two individuals known to each other, the nature of which is influenced by their historical HAIs [4]. The content, quality and pattern of the interactions are important aspects to consider regarding measuring HAR, i.e., the human and the animal getting to know each other. Both the quality and quantity of the interactions influence HAR, with repeated positive, neutral or negative interactions resulting in positive, neutral or negative HAR, respectively [1,2]. A negative HAR can be observed in animals avoiding contact or proximity with humans or showing signs of fear, while conversely animals showing a degree of confidence and lack of fear with people could be deemed as having a positive HAR [1,2]. In a zoo environment it is mostly humans who initiate HAI and influence their quality and quantity [2,5]. According to the agricultural literature positive interactions generally include food provision, grooming and training, whereas negative interactions could include aggression, fast movements, hits, slaps and aversive or unpredictable handling [1,6,7,8]. However, how animals perceive an interaction is also influenced by factors such as past experience [9], personality, species differences [10], motivational state, and their existing relationships with humans [1,2,11]. For example, Waiblinger et al. [10] found that dairy cows that had previously been subject to a handling treatment were less restless and kicked less during a veterinary procedure.

Within the agricultural literature HAR have been extensively studied, often on the basis of assessing an animals’ levels of avoidance and fear. However, such tests usually require direct contact or approach to elicit a response, which is unsuitable for most zoo-housed species in terms of safety and ethics [4]. In addition, studies of stockperson style on farms have shown these to affect the welfare of farm animals [6,12,13]. For example, Ellingsen et al. [12] found that handling calves in a calm and patient manner rather than nervously was associated with a stronger positive mood and fewer handling difficulties. Within the zoo environment keepers generally spend a lot of time with their animals through daily husbandry routines, thus facilitating opportunities for engagement, training and enrichment [14,15,16]. However due to the large variation in species, facilities, husbandry practices, and accessibility, these interactions can vary in quality and quantity [7,17,18,19,20]. For example, Chelluri [16] found that impromptu positive interactions by caretakers resulted in higher levels of agonistic behaviour by chimpanzees (*Pan troglodytes*) and gorillas (*Gorilla gorilla*), suggesting greater arousal and stress in these animals. On the other hand, increased time spent by keepers engaging with a range of felid species (including talking to them) correlated with a likelihood of increased reproductive success, suggesting a positive influence [21]. Most studies in the zoo environment focus on specific quantitative aspects of HAI and indirectly reflect on how this may affect HAR, which may be due to lack of suitable methods [4]. 

Ellingsen et al. [12] argue that qualitative behaviour assessment (QBA), given its sensitivity to subtle expressive cues, is a suitable method for investigating the quality of HAR. QBA is a “whole-animal” methodology which integrates observations of the dynamic emotionally expressive aspects of an animal’s movement and posture (i.e., an animal’s ‘style’ of behaving), using qualitative descriptors such as relaxed, lively, anxious or agitated [22,23]. QBA was originally developed for application to farm animal welfare, and has been extensively tested for reliability and biological validity in a range of farm animal species [24,25,26]. QBA was found to be most effective alongside and complementary to traditional quantitative methods, providing information that can help evaluate the meaning of other measures in terms of the animal’s experience and welfare, and it could be used to study HAR in zoos in similar ways [4]. For example, Minero et al. [27] applied QBA to measure stabled-horse responses due to human contact, and also measured the horses’ response to additional tests (e.g., human approach tests), conducted independently from the QBA. They found that horses perceived as relaxed or at ease showed less avoidance, and responded less aggressively and fearfully to human approach, than horses perceived as uneasy/alarmed. The authors commented that the absence of a fearful and aggressive response would have been difficult to interpret based on quantitative criteria alone, and that using QBA to capture subtly expressive aspects of the horses’ demeanour enabled them to more precisely evaluate the horses’ response. QBA has also been successfully applied to a small group of dogs (n = 10) and so suggests the robustness of the technique in situations [28] that are a common occurrence in zoos.

QBA research in its early stages relied on the use of free-choice profiling (FCP), a method adapted from food science, which allows observers to develop their own descriptors [29], and is particularly suitable for use in new areas of research where it is preferable to ask observers to interpret what they see for themselves, rather than give them pre-fixed descriptors [22]. Given the novel, explorative nature of the current study FCP was felt to be an appropriate choice, avoiding pre-judgment of emotional expression in giraffes. Our hypothesis for the current study was that the use of QBA would help clarify the association between the quality of keeper actions and the emotional response of three giraffes. Further work with larger numbers of animals will be necessary to investigate whether this approach might benefit the study and management of HAR in zoo environments. 

## 2. Materials and Methods 

### 2.1. Ethical Approval

Ethical approval was obtained from Nottingham Trent University for both human and animal components (No. ARE724). Husbandry routines were not changed or affected during data collection and the ARRIVE guidelines were followed where suitable. Prior to video recording, keepers were informed that the study focused on animal behaviour, but were kept unaware that their actions were being observed so as not to alter the way in which they interacted with the animals. The processing of data on keeper behaviour was anonymised therefore no keepers were identifiable throughout the entire study. Consent forms were obtained from all observers involved in the QBA study, which outlined that participants were able to exit the QBA process at any point and their data would be excluded. 

### 2.2. Subjects, Housing and Husbandry 

**Figure 1:**
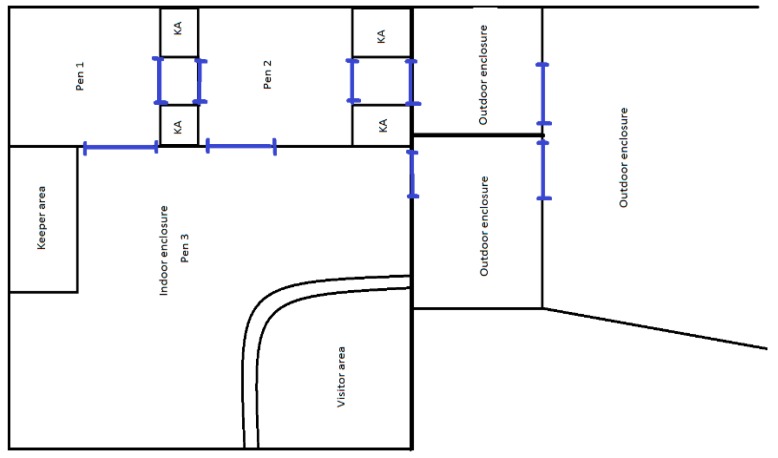
Diagram of giraffe enclosure. Blue lines indicate animal gates operated by keeper staff. All video clips were recorded from the visitor area. KA = keeper area.

A bachelor group consisting of one reticulated giraffe *(Giraffa camelopardalis reticulata)* (giraffe 1 = G1) and two rothschild *(Giraffa camelopardalis rothschildi)* giraffes (giraffe 2 = G2 and giraffe 3 = G3) at a UK zoo were the focus of this study. Giraffes were housed together in an enclosure consisting of an outdoor paddock and indoor area with three pens (Figure 1), and had been housed together at the zoo as a bachelor group for two years, with one week between the arrival of each individual, G3 being the last to arrive at the zoo. Keeper areas were between pen 1 and 2, and between pen 2 and the outdoor enclosure within pen 3. Each pen contained an area with straw bedding, browse hangers, a drinking bowl and a feed bucket. Feeding provisions involved the keepers using ladders to transfer pelleted food from buckets into feeding bowls located on top of fencing in the indoor enclosure, and also attaching browse to fencing. Enclosures were cleaned whilst animals were in neighbouring pens. Although this study did not differentiate between individual keepers, it is worth noting that keeping staff were made up of a total of seven keepers, including both males and females. On one day of the study an unfamiliar human was present gaining work experience as part of the keeper group. 

### 2.3. Data Collection 

#### 2.3.1. Video Collection

Keeper-animal interactions were defined as events initiated by either the keeper or giraffe as categorized in Table 1. All interactions occurred between 14:00–15:00 each day during routine husbandry tasks whilst keepers were in the keeper areas and animals were in the indoor pens.

Video footage of KAI events was obtained over a period of nine days between 28 March and 19 April 2017, filmed from the indoor visitor viewing area using a GoPro Hero 4+ (GoPro, San Mateo, CA, USA) on a tripod. A total of 9 h of footage that included sections where there were no KAIs, were edited into 38 KAI event clips. The clips were selected according to the specific KAIs described in Table 1 and therefore if footage did not include these, it was omitted. Each clip ensured the coverage of the entire KAI event and therefore the length of the video clips varied between 30–120 s. A minimum of 10 clips were obtained for each individual giraffe, distributed across the various KAI events, as shown in Table 2. Due to the infrequent, unpredictable nature of some types of KAI, KAI events could not be recorded in equal numbers for the three individual giraffes. For example, keeper verbal calls were only used when an individual giraffe did not comply with behavioural cues (e.g., gates opening) and therefore the keepers called to the giraffe to encourage them to perform the behaviour. 

#### 2.3.2. Keeper Action Scores 

The KAI events (Table 1) were observed and the extent to which the keeper changed or added to the initial event was documented and scored according to Table 3 (termed keeper action scores). For each KAI event, a cumulative keeper action score was produced, such as for example for the food incentive event whereby the keeper shakes a bucket of food to encourage a giraffe over to a specific area. This would be allocated the initial score of 0, if the keeper then softly called the giraffe’s name the score would increase by 2, if the keeper then started moving suddenly, the score would decrease by 1, if the keeper then shouted towards the giraffe the score would decrease by 2. Therefore, the score would be calculated as 0 + 2, −1, −2 and lead to a cumulative score of −1. 

#### 2.3.3. Qualitative Behaviour Assessment

Eighteen observers participated in the QBA assessment (undergraduate and postgraduate students and lecturers) who had experience in the observation of animal behaviour with various species, but not specifically with giraffes. All observers were given one h of instruction regarding the study context and QBA process, including the opportunity to practice from a few clips of giraffes that were not used in the assessment. Observers were instructed to focus only on the giraffes visible in the clips, and not the keepers, and the sound level in clips was deliberately kept low to minimise any effect of the keepers’ voices and calls. For reasons of availability, observers were split into two groups consisting of 11 and seven people who met at Nottingham Trent University, Brackenhurst campus for a day in May 2018. 

Free-choice profiling (FCP) was used to instruct observers in the QBA assessment of the 38 video clips. FCP is a method originally developed in food science [29] and allows observers to generate their own descriptors and to subsequently use these descriptors for quantitatively scoring animals on a Visual Analogue Scale (VAS). FCP consists of two phases. For phase one observers viewed 15 video clips that had been selected to cover a wide range of giraffe expression in response to KAI. Individual giraffes were not marked for identification in the video clips, but at the start of each clip the focal animal was outlined with a yellow circle. At the end of each clip observers were given two minutes to write down terms that in their view best described the expressive qualities of the focal giraffe in that clip. At the end of the process of watching all 38 clips, each observer had compiled their own set of terms. In phase two, observers were given forms with a VAS placed next to each of their self-generated terms. The VAS were 125 mm long, ranging from minimum (defined as ‘not at all’) to maximum (defined as ‘could not be more’). Observers then viewed each video clip (n = 38) and scored the focal giraffe on each of their own terms, by drawing a vertical line at an appropriate point between ‘minimum’ and ‘maximum’. Observers were given three 10-min breaks during phase two. 

QBA scores were determined by measuring the distance in millimetres between the ‘minimum’ point and the vertical line which the observer had drawn for each term. All scores were inputted into 18 data matrices (one per observer) using Excel files (Microsoft Excel 2017) framed by clip ID and descriptive terms (different for each observer).

### 2.4. Statistical Analysis

#### 2.4.1. Qualitative Behaviour Assessment

The agreement between the 18 individual observer matrices was investigated using the generalized procrustes analysis (GPA), a multivariate statistical technique that is associated with FCP because it does not require the use of fixed variables [29]. GPA can be thought of as a pattern matching mechanism, assuming that even if observers use different variables (terms) for measurement, the distances between measured units (giraffes) will be comparable because these units were the same. As a first step, GPA represents each individual observer data matrix as a multidimensional configuration, in which the number of dimensions correspond to the number of terms used by that observer, and in which the position of the 38 clips is defined by their scores on each term. Equi-dimensionality across data matrices is achieved by adding columns of zeros to individual matrices to match the matrix with the largest number of terms. The observer configurations thus obtained are then matched to each other through a complex iterative process of translation, rotation, reflection and scaling. The final output of this process is the ‘consensus profile’, reflecting a ‘best-fit’ between individual observer scoring patterns (i.e. the average matrix of individual transformed data matrices once no improvement in minimizing inter-configurational distances can be gained by further transformation). The percentage of the total variance between observer configurations explained by this consensus profile, i.e., the degree of inter-observer agreement, is quantified by the so-called Procrustes Statistic (see [30] for a more detailed explanation of these GPA computation steps).

The significance of this consensus profile can be evaluated using a randomization test. Original observer data matrices were analysed in randomized form 100 times, and mean and standard deviation of the ensuing 100 PS values were calculated to reflect a random association value between matrices for each study. A 1-tailed Student-*t*-test (n = 100, df = 99) was then used to determine whether the consensus PS differed significantly from this mean randomized PS. A probability of *p* < 0.001 was taken to indicate that the consensus profile was a meaningful feature of the data set and not a statistical artefact. 

As a second step, principal component analysis (PCA) was applied to reduce the number of dimensions of the GPA consensus profile, and identify the main dimensions of expression explaining the majority of variation between the giraffe clips. Each clip was attributed a score on each of these dimensions, generating two-dimensional ‘giraffe-plots’ showing the distribution of the 38 clips along various combinations of the main dimensions. A standard error ellipse in these plots depicted the reliability of each clip’s position in these frameworks.

In a third interpretative step, the coordinates of the main consensus dimensions were correlated with the coordinates of each of the 18 original individual observer data matrices, creating two-dimensional interpretative ‘word-charts’ for each observer showing the association between that observer’s terms and the main consensus dimensions. The more strongly a term was correlated with a dimension, the more that term could be considered a representative descriptor of that dimension. The two terms showing the highest positive and negative correlations for each principal dimension in each observer word chart were pooled together to create a table of high-loading terms for each consensus dimension. A final step of interpretation for the experimenter was then to summarize this amalgamated information by selecting two or three representative terms as labels for both ends of each of the main consensus dimensions.

The combined analyses of GPA, PCA, PCO, randomisation, correlation, and generation of data plots and word charts were automated through a statistical programme written for Françoise Wemelsfelder by Dr E.A. Hunter at Biomathematics and Statistics Scotland in the UK. 

Two-way analysis of variance and Tukey post-hoc tests were subsequently used to investigate the effects of individual giraffe (n = 3) and KAI event (n = 3) on scores on the main QBA dimensions. The KAI event ‘keeper verbal call’ (KVC) was excluded as they only occurred for one individual. The analysis was carried out using the RStudio statistical software (R version 3.42, R Core Development Team, Vienna, Austria). 

#### 2.4.2. Association between QBA and Keeper Action Scores

Keeper action scores were calculated as described in 2.3.3. To investigate the relationship between keeper action scores and scores on the main QBA dimensions, a spearman rank correlation was conducted. To investigate the difference in keeper action scores between individual giraffes (n = 3) and the KAI event (n = 3), Kruskall–Wallis tests were carried out with Tukey post hoc tests to investigate further differences. The KAI event KVC was excluded from post hoc tests as this KAI event only occurred for one individual. All analyses were carried out using the RStudio statistical software (R version 3.42).

## 3. Results

### 3.1. QBA Analysis

The 18 observers collectively generated a total of 84 descriptive terms, with an average of 19 per observer, ranging from 14–24 terms. The GPA consensus profile explained a significantly higher percentage of the variation between clips (Procrustes statistic: 51.14) than the mean of 100 randomised profiles (mean randomised Procrustes statistic: 33.17 ± 0.09, t_99_ = 58.48, *p* < 0.001), indicating that the observer consensus (i.e., agreement between observers) was a genuine factor of the data set and not a statistical artefact. 

The first two dimensions accounted for the majority of variation between clips (44% and 15%, respectively). Table 4 shows the strongest loading terms, 2 for each observer, on the positive and negative ends of the two QBA dimensions. From this pool of terms, labels were chosen on the basis of three criteria: 1. Reflecting the terms most frequently used by observers, 2. representing additional meanings in the term pool, and 3. where meanings of terms in the pool were very similar, selecting terms with the highest average loading value. On this basis dimension 1 was described as ranging from ‘calm/confident’ to ‘anxious/startled’, and dimension 2 as ranging from ‘impatient/inquisitive’ to ‘calm/distracted’.

### 3.2. Effects of Individual Giraffe and KAI Event on QBA Scores

**Figure 2:**
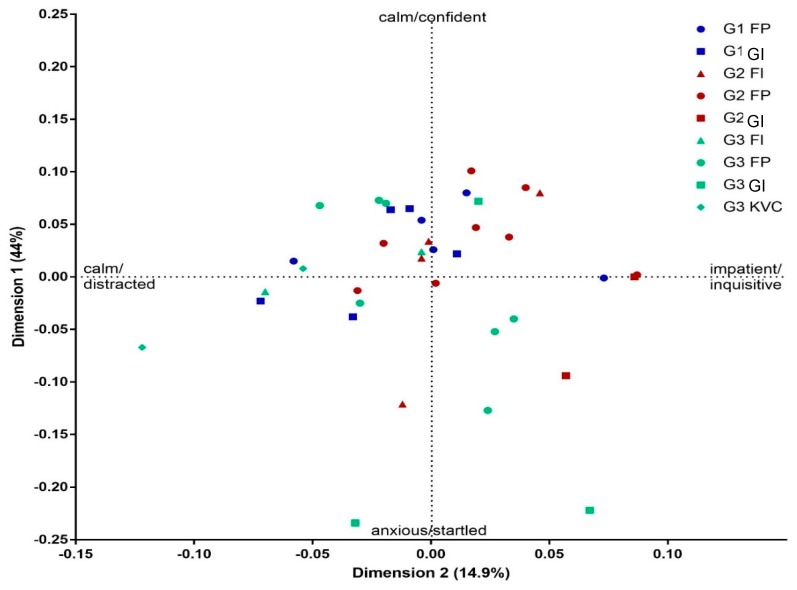
Score plot of video clips labelled to show different Keeper-animal interaction (KAI) events and individual giraffes. The *x* and *y* axis reflect the generalized procrustes analysis (GPA) scaling values for relative differences between video clips on dimensions 1 and 2.

There was no significant difference between individual giraffes (F_2,29_ = 2.430, *p* > 0.05) or KAI events (F_3,29_ = 2.002, *p* = 0.136) in QBA dimension 1 scores. However, there was a significant difference between individual giraffes (F_2,29_ = 4.233, *p* < 0.05; Figure 2) and KAI events (F_3,29_ = 3.878, *p* < 0.05; Figure 2) in QBA dimension 2 scores. Tukey post hoc tests showed a significant difference between individuals G2 and G3 on dimension 2 (*p* < 0.05; Table 5), with individual G2 generally perceived as more ‘impatient/inquisitive’ across KAI events, and individual G3 as more ‘calm/distracted’. There was no significant difference between the KAI events (n = 3) (*p* > 0.05) on QBA dimension 2. Additionally, there was no significant interaction between the individual and KAI event on either of the QBA dimensions.

### 3.3. Association between QBA and Keeper Action Scores

**Figure 3:**
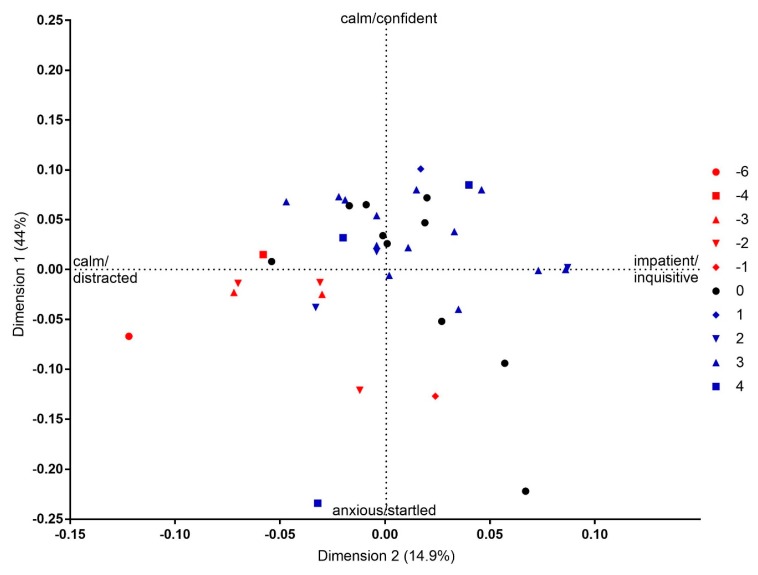
Score plot of video clips labelled by cumulative keeper activity scores. The x and y axis reflect the General Procrustes Analysis scaling values for the relative difference between the assessment of keeper action scores.

A positive correlation was found between keeper action scores and QBA dimension 1 scores (n = 38, rs = 0.40, *p*< 0.05) and QBA dimension 2 scores (n = 38, rs = 0.35, *p* < 0.05), as shown in Figure 3. This indicates that with the increasing quality of keeper action, giraffes were perceived as more ‘calm/confident’ and ‘impatient/inquisitive’, and with decreasing quality as more ‘anxious/startled’ and ‘calm/distracted’. There was no significant effect of either KAI event or individual giraffe on keeper action scores. 

## 4. Discussion

This study applied qualitative behaviour assessment (QBA), using a free-choice profiling method, to investigate the association between KAI events and emotional expression in three giraffes. Eighteen observers showed good agreement in their assessment of the giraffes and established two main dimensions of giraffe expression. Dimension 1 (calm/confident vs anxious/startled) might be interpreted as the assessing level of fear of humans during KAI events. High scores on this dimension might be attributed, for example, to giraffes rapidly pulling their heads away from a keeper during feeding, while low scores might be given to giraffes that are confident with keepers working around them whilst they feed. Dimension 2 (impatient/inquisitive vs calm/distracted) on the other hand might reflect willingness to engage in interaction with humans, such as for example giraffes inquisitively moving or looking towards or away from keepers. However, given the very small sample size of animals in this study, these findings should be regarded as preliminary. Further research with more giraffes is needed to evaluate whether these dimensions have wider relevance.

### 4.1. Individual Differences between Giraffes

On average, the three giraffes were perceived to show similar levels of fear towards humans across KAI events, however the QBA score plot (Figure 2) indicated that individuals G2 and G3 responded more fearfully than G1 to some KAI events. Giraffe G2 was perceived as more impatient/inquisitive than giraffe G3 during KAI events, suggesting this animal was more willing to engage in an interaction with keepers. Differences between individual giraffes were not affected by the type of KAI, or by the quality of keeper action, suggesting there may be underlying factors such as past experience, personality, or social status [1,10,19,20,31] that affect this response. This lack of effect could of course also be due to the small sample size of animals in this study, though it is worth keeping in mind that HAR as a concept, applies to individual animals and needs to always be measured on an individual basis. Studies report varying findings with regard to the effect of such factors on human-animal interactions [1,20,32,33]. For example, Phillips and Peck [34] found that tiger personality did not influence interactions with keepers. However, the personality ratings for tigers did not correlate with behavioural records, so this was possibly due to a short sampling period.

### 4.2. Effects of KAI Type and Keeper Action on Giraffe Expression

The type and quality of KAI an animal experiences will influence how it perceives humans and the intensity of fear it feels for humans [1,2]. Negative keeper actions such as fast sudden movements, speaking loudly and advancing into the flight distance of giraffe, are likely to contribute to the development of negative HAR over time, whereas positive actions such as being calm, fluid movements, speaking softly and providing food, might create more positive HAR. Patel et al. [4] outlined that the HAR is a product of repeated interactions between known humans (keepers) and the animal. Therefore, if fast sudden movements are repeated and the giraffe learns that this is not a threat, the giraffe-keeper relationship could remain neutral, as suggested by Hosey’s model of HAR [2]. However, if the giraffe and keeper have not had such repeated interactions, and a giraffe show signs of avoidance, then the relationship might be negative. Thus, it can be difficult to predict just from physical interactions how giraffes experience relationships with keepers, and this is where QBA can provide helpful information by focusing more closely on subtle expressive clues in the giraffes’ response.

Analysis of the association between keeper action scores and QBA assessments of giraffe expressions indicated that positive keeper actions were linked to calm and confident giraffes with a willingness to interact, whereas negative interactions correlated with more anxious and startled, easily distracted giraffes. These findings are similar to reports in previous studies that link negative interactions and an increased fear of humans [1,2,6,8,35]. For example, Bartnett et al. [36] found that slow movements by stockpersons reduced avoidance behaviour in laying hens, whereas Cransbery et al. [37] reported that movement at speed through a broiler chicken pen increased avoidance of humans. Keeper action scores did not differ across different KAI events or individual giraffes, suggesting other factors were possibly affecting the variation in these scores. One possible factor could have been the presence of different individual keepers during different KAI events. Ward and Melfi [20] showed that an individual keeper’s experience, attitude and knowledge can alter the keeper’s behaviour towards the animals in their care. However, for the present study it was not possible to investigate the contribution of individual keepers to keeper action scores as these were pooled, however this would potentially provide an interesting area for further study using QBA. 

### 4.3. Evaluating the HAR

Giraffe G1 received the most positive/neutral interactions, based on the keeper action scores, and was generally perceived as having low fear and confidence in humans, with a willingness to participate in interactions during KAI events. This suggests a positive HAR with keepers, and supports Hosey’s model of human-animal relationships in zoos [2]. Giraffe G2 generally showed a willingness to interact but a varied level of fear towards humans across KAI events. For example, interactions including GI (giraffe initiated) and FI (food as incentive), which were negative/neutral based on the keeper action score, were associated with this giraffe being perceived as ‘anxious/startled’. Conversely, during interactions that were positive, this individual was perceived more ‘calm/confident’, again supporting the literature [2]. However, in light of this individual showing both positive and negative responses, it could be that there was not yet a well-developed HAR, which could be due to numerous factors such as the individual keepers involved with this giraffe, or the animal’s previous experiences. Giraffe G3 was generally scored as having a high level of fear towards humans and a lower willingness to participate in interactions than individuals G1 and G2. However, when this individual was scored as ‘anxious/startled’ the keeper action score were either positive or neutral, with just one scored negatively. This could suggest that G3, because it was the last giraffe to join this bachelor group, had not yet developed a stable HAR with its keepers, or that the potential KAI pairings recorded for this study were with less familiar keepers. It is not possible to know what sort of prior relationship giraffe G3 had with keepers at its previous collection, however a deliberate increase in positive interactions directed towards this giraffe may reduce the frequency of negative responses. A positive HAR has been associated with positive animal emotions and welfare [1]. This could prove beneficial during daily husbandry and events which may be perceived as aversive by the animal [1,12]. Conversely, a negative HAR could lead to fear responses that could impede welfare and safety for both human and animal. Ensuring repeated positive interactions between keepers and animals could promote positive welfare and ensure a positive HAR in the longer term. A valuable extension to the project reported here could be to ask experienced giraffe keepers to apply QBA to the video footage of giraffes used in this study, and investigate whether this affects any of the results. Zoo animal keepers have been shown to be reliable observers of animal personality and well-being [38], and it would be important to validate the expressive dimensions in giraffes reported here against their expertise.

Finally, it is important to note that this study involved only a small number of giraffes, took place in one location, and recorded a relatively small number of KAIs. It could be therefore that this data lacked wider relevance. However, we would expect that HARs are always unique to the individuals involved, and that therefore giraffes at other institutions, with different keepers and interactions, would be highly likely to respond differently. What this research does support is the usefulness of applying QBA to the measurement of HARs in zoos, which can assist in clarifying individual differences in how animals respond to keeper actions, and the relationships that emerge as a result of these dynamics. This study to our knowledge is the first to document such use of QBA in a zoo setting. 

## 5. Conclusions

The study of HARs and their implications for animal welfare have been extensively investigated in agricultural literature, however there are limited studies within the zoo environment. The combined application of QBA and scoring of keeper action quality allowed individual HARs to be explored for zoo-housed giraffes. Results showed that the quality and type of KAI influenced giraffe behavioural expressions. However, this varied between individual giraffes, suggesting each animal had different perceptions of the KAI events and therefore had developed different HARs. Giraffes that showed a level of fear towards humans could develop compromised welfare, while giraffes showing confidence and a low level of fear could be easier to manage which would be beneficial to their welfare. This is the first application of QBA to the assessment of HARs in zoos. Results of this study, though based on few animals, suggest that the quality of interaction initiated by keepers may influence emotional expression in giraffes. Further research is required to investigate how such interaction may affect longer-term HAR and animal welfare in zoo environments.

## Figures and Tables

**Table 1:** Categories of keeper-animal interaction (KAI) events included within this study.

KAI Event	Code	Description
Giraffe initiated interaction	GI	Giraffe initiated interaction (giraffe approaching keepers through locomotion and/or moving head towards keepers over fences). Keepers present in keeper areas, stationary or cleaning.
Food provision	FP	Keeper using ladder to empty a bucket of pelleted food into feeding bowl located on top of fencing, in indoor enclosure. Keeper using ladder to attach browse to fencing in indoor enclosure.
Food as incentive	FI	Keeper holding and shaking/waving a bucket of pelleted food or browse between keeper area and animal area.
Keeper verbal call	KVC	Keeper verbally calls giraffe from keeper area to encourage desired movement.

**Table 2:** Number of clips obtained for each giraffe (giraffe 1 = G1, giraffe 2 = G2, giraffe 3 = G3) and KAI event (where GI is Giraffe Initiated, FP is food provision, FI is food incentive and KVC is keeper verbal call).

Individual Giraffe	Type of KAI Event	Number of Clips
G1	GI	5
	FP	5
	FI	0
	KVC	0
G2	GI	3
	FP	7
	FI	4
	KVC	0
G3	GI	3
	FP	7
	FI	2
	KVC	2

**Table 3:** Keeper actions and scores used by keepers to slightly alter the initial keeper-animal interaction event to increase or decrease the performance of the desired behaviour. Method adapted for giraffe from Ward and Melfi [19].

Keeper Action Description	Score
Calm, fluid movements (e.g., when providing food)	+1
Providing food to the giraffe (bowl or browse) or speaking softly to giraffe, calling name	+2
Rattling food in front of giraffe (bucket or browse)	+3
No actions described as a (+) or (−) cumulative value	0
Fast sudden movements in vicinity of giraffe	−1
Speaking loudly to giraffe using short words or name	−2
Advancing into the giraffe’s flight distance, e.g. with gesticulating hands or browse	−3
Repetition of any (+) modification each time	+1
Repetition of any (−) modification each time	−1

**Table 4:** Terms showing the highest positive and negative correlations with qualitative behavior assessment (QBA) dimensions 1 and 2 (two per observer). Figures in brackets give the number of observers using that term. Terms highlighted in bold were used to determine dimension labels.

Dimension	Positive Correlation	Negative Correlation
Dimension 1	**Calm (15), relaxed (10), content (4), confident (3)**, bold, curious, patient, unfazed	**Anxious (8), nervous (5), startled (5), skittish (4)**, agitated (2), spooked (2), cautious, evasive, excited, restless, scared, stressed, tense, timid, uneasy, unsure
Dimension 2	**Inquisitive (5), impatient (4), interested (4),** bold (2), curious (2), excited (2), friendly (2), keen (2), active, agitated, attentive, boisterous, demanding, eager, engaged, excitable, frustrated, irritated, restless, startled, stressed	**Calm (7), relaxed (4), alert (2), distracted (2)**, apprehensive (2), cautious (2), focused (2), avoiding, aware, bored, disappointed, distant, docile, hesitant, inquisitive, nervous, passive, reluctant, tense, unsure, vigilant, worried

**Table 5:** Mean ± standard deviation values for individual giraffe scores and Keeper-animal interactions on Qualitative behaviour assessment (QBA) dimensions 1 and 2. G1 = giraffe 1, G2 = giraffe 2, G3 = giraffe 3, GI = giraffe initiated, FP = food provision, FI = food as incentive, KVC = keeper verbal call.

Variable		Mean ± Standard Deviation	
		QBA Dimension 1	QBA Dimension 2
Individual giraffe	G1	0.026 ± 0.04	−0.009 ± 0.03
	G2	0.015 ± 0.06	0.002 ± 0.04 ^a^
	G3	−0.033 ± 0.10	−0.016 ± 0.05 ^a^
KAI event	GI	-0.039 ± 0.10	0.008 ± 0.05
	FP	0.021 ± 0.05	0.007 ± 0.04
	FI	0.004 ± 0.06	−0.008 ± 0.03
	KVC	−0.030 ± 0.04	−0.089 ± 0.03

^a^ = a significant difference between individual giraffes G2 and G3 (F_2,29_ = 4.233, *p* < 0.05; Figure 2)

## References

[1] Waiblinger S., Boivin X., Pederson V., Tosi M., Janczak A.M., Visser E.K., Jones R.B. (2006). Assessing the human–animal relationship in farmed species: A critical review. Appl. Anim. Behav. Sci..

[2] Hosey G. (2008). A preliminary model of human–animal relationships in the zoo. Appl. Anim. Behav. Sci..

[3] Hinde R.A. (1976). Interactions, relationships and social structure. Man.

[4] Patel F., Whitehouse-Tedd K., Ward S.J. (2019). Redefining human-animal relationships: An evaluation of methods to allow their empirical measurement in zoos. Anim. Welf..

[5] Carlstead K. (2009). A Comparative Approach to the study of Keeper-Animal Relationships in the Zoo. Zoo Biol..

[6] Waiblinger S., Menke C., Coleman G. (2002). The relationship between attitudes, personal characteristics and behaviour of stockpeople and subsequent behaviour and production of dairy cows. Appl. Anim. Behav. Sci..

[7] Hemsworth P.H., Barnett J.L., Coleman G.J. (1993). The human-animal relationship in agriculture and its consequences for the animal. Anim. Welf..

[8] Hemsworth P.H. (2003). Human–animal interactions in livestock production. Appl. Anim. Behav. Sci..

[9] Anderson U.S., Benne M., Bloomsmith M.A., Maple T.L. (2002). Retreat space and human visitor density moderate undesirable behaviour in petting zoo animals. JAAWS.

[10] Waiblinger S., Menke C., Korff J., Bucher A. (2004). Previous handling and gentle interactions affect behaviour and heart rate of dairy cows during a veterinary procedure. Appl. Anim. Behav. Sci..

[11] Munksgaard L., De Passillé A.M., Rushen J., Thodberg K., Jensen M.B. (1997). Discrimination of People by Dairy Cows Based on Handling. J. Dairy Sci..

[12] Ellingsen K., Coleman G.J., Lund V., Mejdell C.M. (2014). Using qualitative behaviour assessment to explore the link between stockperson behaviour and dairy calf behaviour. Appl. Anim. Behav. Sci..

[13] Hemsworth P.H., Coleman G., Barnett J.L., Borg S. (2000). Relationship between human-animal interactions and productivity of commercial dairy cows. J. Anim. Sci..

[14] Carrasco L., Colell M., Calvo M., Abello M.T., Velasco M., Posada S. (2009). Benefits of training/playing therapy in a group of captive lowland gorillas (*Gorilla gorilla gorilla*). Anim. Welf..

[15] Claxton A.M. (2011). The potential of the human–animal relationship as an environmental enrichment for the welfare of zoo-housed animals. Appl. Anim. Behav. Sci..

[16] Chelluri G.I., Ross S.R., Wagner K.E. (2013). Behavioral correlates and welfare implications of informal interactions between caretakers and zoo-housed chimpanzees and gorillas. Appl. Anim. Behav. Sci..

[17] Perlman J.E., Bloomsmith M.A., Whittaker M.A., McMillan J.L., Minier D.E., McCowan B. (2012). Implementing positive reinforcement animal training programs at primate laboratories. Appl. Anim. Behav. Sci..

[18] Bloomsmith M.A., Stone A.M., Laule G.E. (1998). Positive reinforcement training to enhance the voluntary movement of group-housed chimpanzees within their enclosures. Zoo Biol..

[19] Ward S.J., Melfi V. (2013). The implications of husbandry training on zoo animal response rates. Appl. Anim. Behav. Sci..

[20] Ward S.J., Melfi V. (2015). Keeper-Animal Interactions: Differences between the Behaviour of Zoo Animals Affect Stockmanship. PLoS ONE.

[21] Mellen J.D. (1991). Factors influencing reproductive success in small captive exotic felids (Felis spp.): A multiple regression analysis. Zoo Biol..

[22] Wemelsfelder F., Hunter E.A., Mendl M.T., Lawrence A.B. (2001). Assessing the ‘whole animal’: A Free-Choice-Profiling approach. Anim. Behav..

[23] Wemelsfelder F. (2007). How animals communicate quality of life: The qualitative assessment of animal behaviour. Anim. Welf..

[24] Fleming P.A., Clarke T., Wickham S.L., Stockman C.A., Barnes A.L., Collins T., Miller D.W. (2016). The contribution of qualitative behavioural assessment to appraisal of livestock welfare. Anim. Prod. Sci..

[25] Wemelsfelder F., Mullan S., Mellor D.J., Bayval A.C.D. (2014). Applying ethological and health indicators to practical animal welfare assessment. Animal Welfare: Focusing on the Future.

[26] Rutherford K.M.D., Donald R.D., Lawrence A.B., Wemelsfelder F. (2012). Qualitative Behavioural Assessment of emotionality in pigs. Appl. Anim. Behav. Sci..

[27] Minero M., Dalla Costa E., Dai F., Canali E., Barbieri S., Zanella A., Pascuzzo R., Wemelsfelder F. (2018). Using qualitative behaviour assessment (QBA) to explore the emotional state of horses and its association with human-animal relationship. Appl. Anim. Behav. Sci..

[28] Walker J., Dale A., Waran N., Farnworth M., Clarke N., Wemelsfelder F. (2010). The assessment of emotional expression in dogs using a free choice profiling methodology. Anim. Welfare.

[29] Oreskovich D.C., Klein B.P., Sutherland J.W., Lawless H.T., Klein B.P. (1991). Procrustes Analysis and its applications to free-choice and other sensory profiling. Sensory Science: Theory and Applications in Foods.

[30] Wemelsfelder F., Hunter E.A., Mendl M.T., Lawrence A.B. (2000). The spontaneous qualitative assessment of behavioural expressions in pigs: First explorations of a novel methodology for integrative animal welfare measurement. Appl. Anim. Behav. Sci..

[31] Rousing T., Waiblinger S. (2004). Evaluation of on-farm methods for testing the human–animal relationship in dairy herds with cubicle loose housing systems—Test–retest and inter-observer reliability and consistency to familiarity of test person. Appl. Anim. Behav. Sci..

[32] Rossman Z.T., Padfield C., Young D., Hart L.A. (2017). Elephant-initiated interactions with humans: Individual differences and specific preferences in captive African elephants (Loxodonta africana). Front. Vet. Sci..

[33] Wielebnowski N.C. (1999). Behavioral differences as predictors of breeding status in captive cheetahs. Zoo Biol. Publ. Affil. Am. Zoo Aquar. Assoc..

[34] Phillips C., Peck D. (2007). The effects of personality of keepers and tigers (Panthera tigris tigris) on their behaviour in an interactive zoo exhibit. Appl. Anim. Behav. Sci..

[35] Breuer K., Hemsworth P.H., Coleman G.J. (2003). The effect of positive or negative handling on the behavioural and physiological responses of nonlactating heifers. Appl. Anim. Behav. Sci..

[36] Barnett J.L., Hemsworth P.H., Hennessy D.P., McCallum T.H., Newman E.A. (1994). The effects of modifying the amount of human contact on behavioural, physiological and production responses of laying hens. Appl. Anim. Behav. Sci..

[37] Cransberg P.H., Hemsworth P.H., Coleman G.J. (2000). Human factors affecting the behaviour and productivity of commercial broiler chickens. Br. Poult. Sci..

[38] Whitham J.C., Wielebnowski N. (2009). Animal-based welfare monitoring: Using keeper ratings as an assessment tool. Zoo Biol..

